# Understanding the Impact of Hazardous and Harmful Use of Alcohol and/or Other Drugs on ARV Adherence and Disease Progression

**DOI:** 10.1371/journal.pone.0125088

**Published:** 2015-05-01

**Authors:** Rehana Kader, Rajen Govender, Soraya Seedat, John Randy Koch, Charles Parry

**Affiliations:** 1 Alcohol, Tobacco and other Drug Research Unit, Medical Research Council, Cape Town, South Africa; 2 Centre for Social Science Research and Department of Sociology, University of Cape Town, Cape Town, South Africa; 3 Department of Psychiatry, University of Stellenbosch, Stellenbosch, South Africa; 4 Institute for Drug and Alcohol Studies, Virginia Commonwealth University, Richmond, Virginia, United States of America; Rega Institute for Medical Research, BELGIUM

## Abstract

The objective of this study was to understand the impact of hazardous and harmful use of alcohol and/or other drugs on ARV adherence and disease progression among HIV patients. A cross-sectional study design was used. A total of 1503 patients attending HIV clinics in Cape Town, South Africa were screened for problematic substance use. A sub-sample of 607 patients (303 patients who screened positive for problematic substance use and 304 who did not) participated in this study. Hazardous or harmful alcohol use and problematic drug use predicted missing and stopping ARVs which, in turn, was associated with a decrease in CD4 counts and more rapid HIV-disease progression and poorer health outcomes in people living with HIV/AIDS (PLWHA). The findings of this study underscore the need for an integrated approach to managing substance-use disorders in PLWHA.

## Introduction

The human immunodeficiency virus (HIV) and acquired immunodeficiency syndrome (AIDS) have become a global pandemic and a major health challenge, especially in sub-Saharan Africa. Substance use, like HIV, is another major health challenge facing these countries, particularly South Africa. Since South Africa’s first democratic elections in 1994 and the country’s re-entry into the global economy, there has also been an increase in drug trafficking. Although alcohol continues to be the primary and most abused substance in South Africa, in recent years there has been an increase in the use of heroin, cocaine and amphetamine-type stimulants [1].

In the Western Cape particularly, for the reporting period July-December 2013, 86% of all admissions for substance abuse treatment were for methamphetamine (‘tik’), alcohol, cannabis and heroin. Methamphetamine was reported in 33% as a primary substance of abuse, cannabis was reported as common primary substance of abuse in 25% of admissions and Mandrax was reported in 21% and heroin in 13% of admissions in the Western Cape [2].

According to a national survey conducted in south Africa, overall 9.6% of South Africans engaged in past-month binge drinking (4 or more drinks for females and 5 or more drinks for males on one occasion) and 9% of the sample (men and women) was found to engage in hazardous or harmful use of alcohol as defined by the AUDIT. This study concluded that there was an increase in the prevalence of current, binge, and hazardous or harmful drinking from 2005 to 2008 in South Africa [3]. In South Africa one unit of drink contains 12 grammes of alcohol [4]. Levels of binge drinking (among drinkers) particularly high in South Africa compared to other African countries [5].

There is overwhelming evidence in the literature regarding the relationship between substance use and HIV status [6]. In particular, studies have found high rates of substance use among people who are HIV positive [7,8]. The types of substances referred to in such studies range from alcohol to illicit drugs such as heroin, cocaine, marijuana, stimulants and other drugs.

Most of this research has centred on substance use as a risk factor for contracting HIV. However, recent research has focused on the impact of alcohol consumption in people who have already contracted the virus. Hazardous or harmful use of alcohol in people living with HIV/AIDS (PLWHA) was found to be associated with poor adherence to ARVs, re-infection, decreased viral suppression, increased viral replication and earlier onset of death [9,10].The combined hazardous use of alcohol and drugs was also associated with the lowest odds of ARV use adherence and viral suppression [11]. In a similar vein, stimulant use among PLWHA was found to predict faster HIV disease progression, decrease in CD4 counts and AIDS related mortality [12].

Taken together, these studies provide strong evidence for the role of substance abuse in advancing disease progression by diminishing adherence to the ARV regimen. Furthermore, the impact on ARV adherence by alcohol and drug abuse is both singular and collective, with the combined effect leading to significantly poorer health outcomes.

However, in South Africa, few studies have focused on the relationship between alcohol abuse and poor ARV adherence [13,14]. This is the first study that investigated both alcohol and drug abuse and its relationship to ARV adherence in South Africa. The objectives of this study were to determine the predictors of hazardous or harmful use of alcohol and problematic drug use, and the impact of these on ARV adherence and CD4 counts.

## Materials and Methods

A cross-sectional study design was used. The power calculation for this study indicated that a sample size of 1 503 participants was needed. A total of 1503 patients attending 15 HIV clinics in Cape Town, South Africa were screened for alcohol and drug use, using the Alcohol Use Disorders Identification Test (AUDIT) and/or on the Drug Use Disorders Identification Test (DUDIT).For further sub analyses a significance level of 0.025 and power of 90% were specified. This yielded an optimal sample size of 216 substance abusers. To boost the sub-analyses of data, we included 303 substance abusers (i.e. patients who scored above the cut-off score of 8 for both men and women on the AUDIT and a cut-off score of 6 or more for men, and a cut-off score of 2 points for woman was used for the DUDIT) and a comparison group of 304 non-substance abusers (i.e. patients who scored below the cut-off score on the AUDIT and DUDIT) at the eight clinics. The selection of these 607 participants from the 1503 to participate further comprised the first 38 patients selected randomly at each of the eight clinics who were substance abusers and the first 38 patients who were not substance abusers. The sample was drawn from clinics deemed by the Department of Health to be representative of all state funded HIV clinics in the metropole.

Patients attending these HIV clinics were informed of the study by their attending clinician (nurse, doctor, HIV counsellor). If patients showed an interest in participating in the study, they were referred to the trained fieldworkers for this study.

All participants completed written informed consent forms prior to participation in the study, and these forms were stored as official record of the informed consent. The consent procedure was approved by Human Research Ethics Committee of the Health Science Faculty of the Stellenbosch University, which also approved the entire study, ref no: N08/10/294. The committee thus considered the patient information procedure ethically adequate for this study. Questionnaires were administered in the participant’s language of choice (Afrikaans, Xhosa and English) and all translations were done using the double blind technique. Participants who were found on the AUDIT and/or DUDIT to be possibly alcohol or drug dependent were referred for treatment.

### Instruments

#### Socio-demographic variables and health characteristics

A brief questionnaire was used to collect relevant demographic information (age, race, gender, etc.) from the participant and clinical records were used to obtain clinical information (date of diagnosis, date of commencement of ARVs, date of diagnosis of tuberculosis (TB) and opportunistic infections, etc.). As used in this study, race (the categories of “white", "black", and "Coloured”) refers to demographic markers and does not denote any inherent characteristics. Their continued use in South Africa is important for monitoring improvements in health and socio-economic disparities, identifying vulnerable sections of the population, and planning effective prevention and intervention programmes.

#### The Alcohol Use Disorders Identification Test (AUDIT)

The AUDIT was developed by the World Health Organisation and is used to screen people with hazardous and harmful patterns of alcohol use and alcohol dependence. The AUDIT is scored as follows: 0–7 indicates abstinence or low-risk drinking; 8–15 is indicative of hazardous drinking patterns; 16–19 indicates harmful levels of drinking and 20–40 indicates possible dependence [15]. The Cronbach alpha for the AUDIT was 0.81 for this sample.

#### The Drug Use Disorders Identification Test (DUDIT)

The DUDIT is a screening instrument used to screen people with problematic drug use. A score of 6 or more indicates problematic drug use in men and a score of 2 or more indicates the same in women. For both genders, a score 25 or more indicates a high probability of dependence on one or more drugs [16].The Cronbach alpha for the DUDIT was 0.76 for this sample.

#### ARV Adherence Assessment

Two instruments were used to assess ARV adherence, the Morisky Scale and the ARV Adherence Questionnaire. The Morisky Scale is a self-report measure shown to have sound concurrent and predictive validity [17]. The ARV Adherence Questionnaire was used as adapted in a previous South African study [18].The questionnaire assesses various reasons for missing ARV doses and stopping ARVs. ARV adherence was measured in terms of missing ARVs and stopping ARVs. Missing ARVs comprised not taking ARVs for the following reasons: they did not want others to see they were taking medication, they were away from home, they just forgot, because they were busy with other things. Stopping ARVs was defined as participants stopping their medication for any of a number of reasons, e.g., because they felt better, felt sick or depressed, felt overwhelmed by the number of pills, worried about getting side effects from medication, actually getting side effects from medication and because they ran out of pills. The Cronbach alpha for the Morisky Scale was 0.62 and for the ARV Adherence Questionnaire it was 0.73 for this sample.

### Data Analysis

All data was analysed using SPSS, version18. A 95% confidence interval and a 5% level of significance were used to interpret statistical significance. All statistical tests were two-tailed. Descriptive analyses (means, standard deviations and frequencies) were used to describe demographic data and clinical characteristics.

Univariate tests of association for categorical variables (chi-square tests) were used to examine associations between problematic drug use and gender, ARV adherence (stopping, missing a dose, being careless and forgetting to take ARVs). The t-test was used to test for group differences on hazardous or harmful use of alcohol and across gender and ARV adherence. Pearson correlations were employed to assess the relationship between hazardous or harmful use of alcohol and CD4 count. Multivariate linear relationships were explored using regression analysis.

Following the regression analysis, a path analysis model was developed to examine 1) the determinants of hazardous or harmful alcohol and/or problematic drug use, and 2) the impact of these on ARV adherence and CD4 counts. Variables for the model were selected on the basis of the bivariate and regression analyses.

## Results

### Descriptive and Bivariate Analysis

#### Demographic characteristics

The sample consisted almost entirely of Black (89%) and Coloured (10%) participants reflecting the demographics of the population of PLWHA attending the public clinics were data collection occurred. The mean age of participants was 35.9 years. Most participants were female (67.5%), single (64.1%), unemployed (66.7%), and had some high-school education (62.3%). The monthly income for most of the participants (62.4%) was less than R1 000 (US$120) and 35% earned between R1 000 and R5 000 per month (US$120 and US$602) (Table 1).

**Table 1:** Demographic characteristics of the sample.

MEAN AGE (35.91) SD = 8.0		
GENDER	n	%
Male	197	32.5
Female	410	67.5
RACE		
Black	541	89.1
Coloured	62	10.2
Other	4	0.7
CURRENT MARITAL STATUS		
Single	389	64.1
Widowed	25	4.1
Separated	19	3.1
Divorced	13	2.1
Married or living with a significant other	161	27.5
CURRENT LIVING SITUATION		
Live alone	81	13.3
Live with other adults, no children	122	20.1
Live with other adults and children	285	47.0
Live with children	119	19.6
HIGHEST LEVEL OF EDUCATION COMPLETED		
No formal education	49	8.1
Completed primary school	102	16.8
Attended high school but did not complete matric (Grade 12)	378	62.3
Completed matric (Grade 12)	69	11.4
Attended university, college or technikon but did not graduate	4	0.7
Graduated from university, college or technikon	5	0.8
CURRENT MARITAL STATUS		
Single	389	64.1
Widowed	25	4.1
Separated	19	3.1
Divorced	13	2.1
Married or living with a significant other	161	27.5
CURRENT LIVING SITUATION		
Live alone	81	13.3
Live with other adults, no children	122	20.1
Live with other adults and children	285	47.0
Live with children	119	19.6
CURRENT WORK SITUATION		
Employed full time	99	16.3
Employed part time	85	14.0
Student	7	1.2
Unemployed	405	66.7
Disabled	9	1.5
Homemaker	1	0.2
Retired	1	0.2

#### Health status

The average time between diagnosis of HIV and data collection was 3.8 years. The current mean CD4 count was 305.5 and the CD4 nadir (lowest CD4 count in the previous 12 months) was 264.8. The majority of the participants, 539 (88.8%) were on ARVs.

#### Hazardous or harmful use of alcohol and problematic drug use

Just under half of all participants (46%) scored at or above the clinically significant cut-off score of ≥8 on the AUDIT, indicating the likelihood of hazardous or harmful drinking, and 15% scored above the respective cut-off points on the DUDIT, indicating problematic drug use.

#### Gender Differences

There were significant gender differences in the hazardous or harmful use of alcohol, with males being significantly more likely than females to engage in hazardous or harmful use of alcohol (t = 4.988, *p* = 0.00). Similarly, there were significant gender differences in problematic drug use (x^2^ = 43.185, *p* = 0.00), and males were three times more likely to be problematic drug users than females. Moreover, there was a significant association between gender and missing ARV doses (x^2^ = 6.326, *p* = 0.01). More males (48.2%) than females (36.8%) reported missing a dose of ARVs. There was a significant association between gender and CD4 counts (t = -2.825, *p* < 0.00) with females having significantly higher CD4 counts than males.

#### ARV adherence

There was a significant association between hazardous or harmful use of alcohol and stopping ARVs (t = -5.364, *p* = 0.00), missing ARV doses (t = -7.737, *p* = 0.00), forgetting to take ARVs (t = 6.264, *p* = 0.00), and being careless about taking ARVs (t = 7.773, *p* = 0.00). Participants reporting hazardous or harmful use of alcohol were significantly more likely to either stop, miss, forget or be careless about taking their ARVs than their counterparts without alcohol problems.

There was a similarly significant association between problematic drug use and stopping ARVs (x^2^ = 5.915, *p* = 0.05), missing ARV doses (x^2^ = 31.794, *p* = 0.00), forgetting to take ARVs (x^2^ = 13.987, *p* = 0.00), and being careless about taking ARVS (x^2^ = 19.852, *p* < 0.05). Participants with problematic drug use were much more likely to stop their ARVs and were three times more likely to miss taking their ARVs than participants without drug problems. Problematic drug users were also almost twice as likely to forget to take their ARVs or be careless about taking them than their counterparts without these drug problems.

#### CD4 Counts

There was a significant negative correlation between AUDIT total scores and CD4 counts: as the AUDIT total scores increased, CD4 counts decreased (r = -0.106, *p* = 0.01). There was no significant correlation between DUDIT scores and CD4 counts (t = 0.935, p = 0.35).

### Logistic Regression Analysis

As a precursor to the path analysis model logistic regression analysis was conducted to examine the impact of various demographic and substance abuse behaviours on ARV adherence. The results of this analysis are presented in Table 2 and Table 3. For the purposes of the logistic regression and path analysis, the employment variable was recoded into a binary variable (Employed/Unemployed), while gender and age were treated as originally measured. The remaining demographic variables were not entered into the logistic regression and path analysis modelling as a result of lack of bivariate statistical significance between each of these variables and AUDIT and DUDIT.

**Table 2:** Determinants of Missing ARVs—Logistic Regression Analysis.

Missed medication ^a^		B	Sig.	Exp(B)	95% Confidence Interval for Exp(B)	
					Lower Bound	Upper Bound
Missed medication	Intercept	-1.343	.006			
	AGE	-.010	.417	.990	.965	1.015
	AUDIT	.055	.000	1.057	1.037	1.076
	DUDIT	.558	.000	1.747	1.298	2.350
	Male	.062	.779	1.064	.689	1.645
	Female	0	.	.	.	.
	Employed	.270	.198	1.310	.868	1.975
	Unemployed	0	.	.	.	.

^a^ The reference category is: Never missed medication.

**Table 3:** Determinants of Stopping ARVs—Logistic Regression Analysis.

Stopped Medication ^a^		B	Sig.	Exp(B)	95% Confidence Interval for Exp(B)	
					Lower Bound	Upper Bound
Stopped medication	Intercept	-1.637	.006			
	AGE	-.012	.433	.988	.958	1.019
	AUDIT	.052	.000	1.054	1.031	1.076
	DUDIT	.100	.536	1.105	.805	1.518
	Male	-.052	.849	.950	.559	1.613
	Female	0	.	.	.	.
	Employed	-.290	.287	.748	.439	1.276
	Unemployed	0	.	.	.	.

^a^ The reference category is: Never stopped medication.

The logistic regression analysis revealed that AUDIT total scores was the only significant determinant of stopping ARVs, and both AUDIT and DUDIT total scores were significant determinants of missing ARVs. The lack of statistical significance for the demographic variables was not surprising as it was hypothesized, as will be seen later in the path analysis model, that these variables would be directly significant in determining substance abuse behaviours, and that these substance abuse behaviours would in turn be direct determinants of ARV adherence.

### Path Analysis Modelling

**Fig 1:**
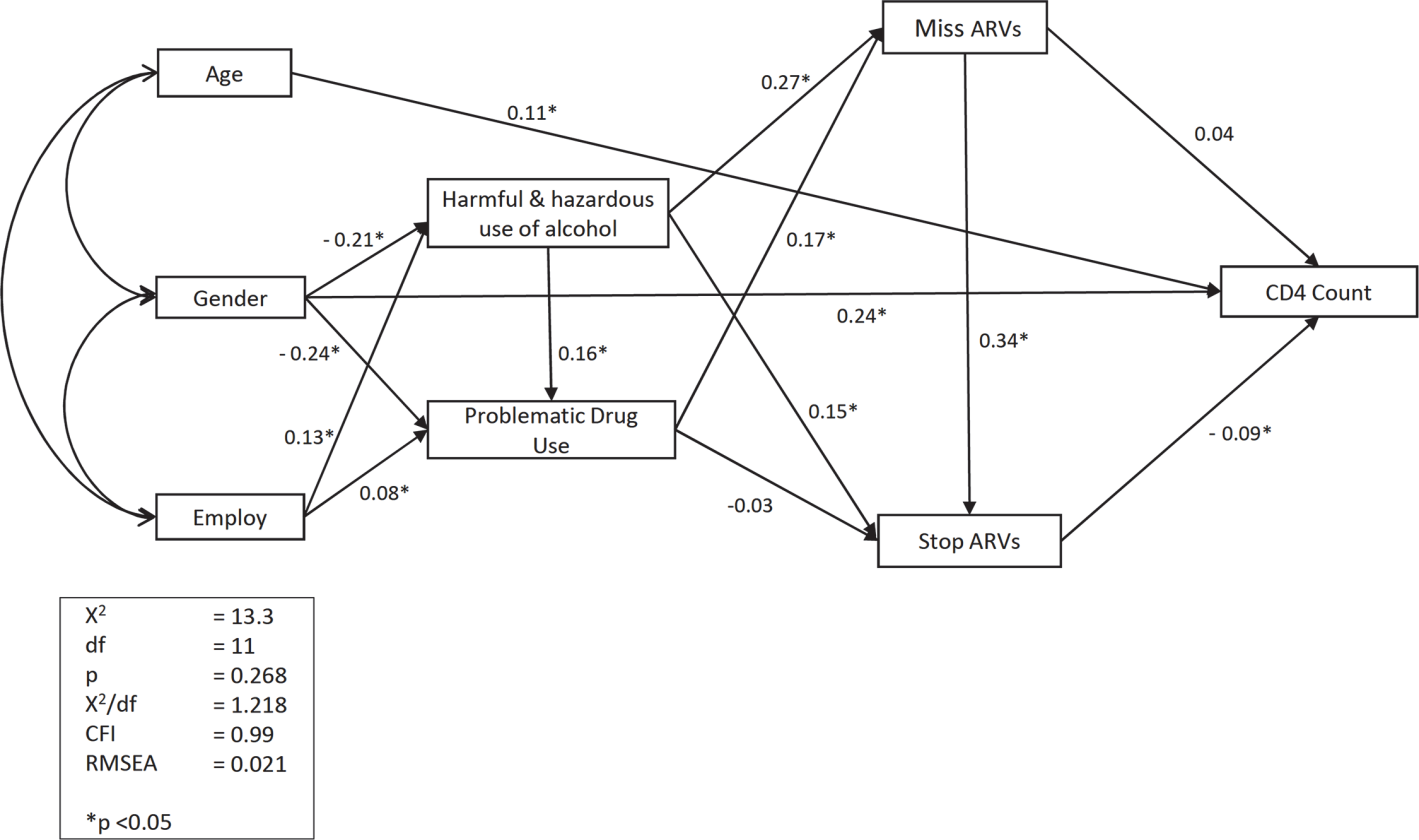
Modelling determinants of ARV adherence and health status.

To test for the simultaneous impact of the various demographic and substance abuse variables on ARV adherence and CD4 counts, a path analysis model was specified and tested (Fig 1). As the model featured ARV adherence data, participants who were not on ARVs were excluded from the analysis. The results of the path analysis modelling revealed that there were no residual errors and no data problems with the measurement model, thereby permitting the use of model indices and the interpretation of model results. The output indicated that the structural model provided a good fit to the data, as indicated by the following indices:

- CFI = 0.99 (a CFI of 0.90 and above is regarded as a well-fitting model)
- Root Mean Square Error of Approximation (RMSEA) = 0.021 (a RMSEA value of 0.03 and 0.05 is regarded as evidence of a good fitting model)
- *χ* ^2^ = 13.3 with *df* = 11 and *χ* ^2^/*df* ratio of 1.218 (a *χ* ^2^/*df* ratio of 1 to 4 is considered as evidence of a good model).

Furthermore, the Lagrange Multiplier Tests outputs confirmed the validity of the good fit indices and indicated that no further modifications were necessary to the model.

Looking more closely at the tested model, a number of significant direct predictive pathways were identified:

1. Gender was found to be significant in predicting the harmful and hazardous use of alcohol (ß = - 0.21, p = 0.00) and drugs (ß = - 0.24, p = 0.00), with males registering significantly higher levels of problematic substance use than females.
2. Gender (ß = - 0.24, p = 0.00) and age (ß = 0.11, p = 0.01) were found to be significant in directly predicting CD4 counts: being male and of younger ages was predictive of significantly lower CD4 counts.
3. Unemployment was found to be a direct determinant of alcohol use (ß = 0.13, p = 0.00) and drugs (ß = 0.08, p = 0.05), with the unemployed being significantly more likely to engage in the harmful and hazardous use of alcohol and drugs.
4. Harmful and hazardous use of alcohol (ß = 0.16, p = 0.00) was a direct determinant of problematic drug use: those who indicated harmful and hazardous use of alcohol were more likely to engage in problematic drug use.
5. Harmful and hazardous use of alcohol was a direct determinant of missing ARVs (ß = 0.27, p = 0.00) and stopping ARVs (ß = 0.15, p = 0.00), while problematic drug use was a significant determinant of missing ARVS (ß = 0.17, p = 0.00) but not stopping ARVs (ß = -0.03, p = 0.49).
6. Missed ARVs was found to predict stopping ARVs (ß = 0.34, *p* = 0.00 participants who missed taking their ARVs were more likely to stop taking their ARVs.
7. Stopping ARVs (ß = -0.09, *p* = 0.04) significantly predicted CD4 counts: those who stopped taking their ARVs had significantly lower CD4 counts.

## Discussion

The results indicate that harmful and hazardous use of alcohol and problematic drug use are statistically significant and important determinants of ARV adherence, and consequently, of health outcomes as measured by CD4 counts. In terms of determining patterns of substance abuse, gender and unemployment were found to be significant in terms of determining substance abuse, with males and the unemployed being more likely to engage in such behaviours.

In terms of ARV adherence, substance abuse has nuanced effects. The harmful and hazardous use of alcohol was found to have direct significant impacts on all forms of ARV adherence (missing and stopping ARVS), while problematic drug use was found to only impact on missing and not stopping ARVS. However, those who missed their ARVS were more likely to also stop them, indicating an indirect effect for problematic drug use. This is consistent with findings from a study that reported that hazardous drinkers, heavy drinkers and binge drinkers were more likely to miss a dose of medication or to take medication off-schedule [7].Similarly, another reported that hazardous or harmful drinkers of alcohol were nine times more likely to not adhere to ARVs than their non-drinking counterparts [19].This is consistent with a study that reported alcohol users were over five times more likely to be non-adherent compared to participants who did not use alcohol [20]. In a meta-analysis of the association between alcohol use and ARV adherence alcohol users were 50%-60% as likely to be adherent to ARVs compared to those who abstained from alcohol or drank relatively less [10].Several factors moderated the association between alcohol and ARV adherence in this study, such as a higher proportion of men being non-adherent, which is consistent with the findings of this study.

The prevalence of hazardous and harmful use of alcohol in this study is much higher than was previously documented in studies conducted in sub-Saharan Africa where hazardous or harmful use of alcohol was reported to range between 2.6% to 24.3% in PLWHA [21,22,23,24].Similarly, studies in South Africa have reported lower rates of alcohol abuse/dependence in PLWHA than in the current study, ranging from 7% to 12.9% but this could be partly due to differing measuring approaches [25,26,27].

Furthermore, hazardous or harmful alcohol use was a direct predictor of problematic drug use, which in turn, predicted poorer adherence and lower CD4 counts. These findings are consistent with the findings of a study in which problem drinking was reported to be associated with illicit drug use and binge drinking and non-adherence to HAART [28].Similarly, other studies have documented that active drug users underutilized HAART compared to former and non-drug users and had sub-optimal virologic and immunologic responses to antiretroviral therapy [29].The rate of problematic drug use (15%) is comparable to a study that reported that 9% of PLWHA met the criteria for drug dependence in the previous 12 months and 10% abusing drugs [30].This rate is considerably lower than international studies where current drug use in PLWHA is in the order of 46%-64% [31,32].

Gender and unemployment were found to be significant determinants of substance use with more males reporting hazardous or harmful use of alcohol and/or drugs. Being male and of a younger age were also found to be a significant determinant of lower CD4 counts. These findings are further substantiated by studies conducted in South Africa examining the role of gender, age, unemployment and other socio-demographic factors in determining problematic substance use and ARV poor adherence [25,26,33].

The study found that harmful and hazardous use of alcohol and/or drugs has an impact on ARV adherence. ARV adherence impacts on diminished health status, i.e., lower CD4 counts, only in the most extreme version where ARVs were stopped, while the less extreme version of non-adherence, i.e., missing ARVs did not have a similar significant effect on CD4 counts. Taken together, this suggests that patients who stop ARVs are at greatest risk for a higher viral load, while those who miss their ARVs are at less risk, though this risk could escalate as missing ARVs could easily also lead to actually stopping them.

There are a number of limitations that deserve mentioning. Firstly, the sample was drawn from the Cape Metropole area and may not be representative of the HIV-treatment seeking population across South Africa with regards to patterns of substance use and ARV adherence. Secondly, the reporting of alcohol and drug use was measured by self-reports and not confirmed by laboratory testing for alcohol and drug abuse. However, it should be noted that a pilot study among PLWHA comparing self-report alcohol and drug use with urine and hair tests reinforced the value of self-report data on alcohol and drugs [34].Thirdly, the reporting of ARVs was also measured by self-reports and not confirmed by objective biological markers. Study findings have revealed that patients’ self-reporting on ARV adherence tend to overestimate adherence when compared to objective measures, such as electronic monitoring [35].Therefore, a more standardised and objective method of assessing ARVs should be considered that will provide a more accurate measurement of ARV adherence in future.

Fourthly, the multivariate models are limited in that they only account for the impact of variables selected for inclusion in the analysis. This implies that further models employing other determinant variables—demographic or behavioural—might lead to different results. For instance, the impact of substance abuse on ARV adherence may be mediated or moderated by the presence of co-occurring psychiatric conditions such as depression, anxiety and post traumatic stress disorder. These factors may also play a role in directly impacting the clinical status of patients receiving ARVs. However, the purpose of the research was not to examine an exhaustive list of likely determinants, but rather to clarify the relative strength of determination of the identified variables. Despite these limitations, this study makes an important contribution in identifying multiple predictors of ARV adherence and CD4 count and the association with health outcomes of PLWHA. However, of importance and to our knowledge, this is the first large systematic investigation of problematic alcohol and drug use in a representative sample of persons receiving treatment from HIV clinics in South Africa. Therefore, it can form the basis for replication studies in other parts of the country, including more longitudinal studies of PLWHA.

## Conclusion

The findings of this study underscore the need for an integrated approach to managing substance-use disorders in PLWHA. Screening for substance use in PLWHA should be standard practice and conducted routinely at primary health-care centres. The integration of substance use and HIV services should be considered to ensure that patients are seen at a single facility. Cross-training staff at HIV and substance abuse centres should be considered. Cross-training should focus on the link between substance abuse, mental health problems and HIV/AIDS, and on screening. Primary health- care workers should also be cross-trained to identify these disorders and refer appropriately where necessary [36].

Screening instruments are cost effective and can be easily administered by any health professional required they are given the proper training. For example, when using the AUDIT as a screening instrument; patients who are screened as low-risk drinkers should be given information about the risks of drinking. Medium-risk drinkers could be given simple advice, brief counselling and their drinking monitored at follow-up visits and patients who are high-risk drinkers could be referred to a specialist for further diagnosis and treatment [15]. In addition, treatment providers should inquire about drug use and poly-drug use before prescribing ARVs [37].

It could also help considering using some form of Daily Observed Treatment System (DOTS) to improve adherence, that could be rolled out to patients at primary health-care facilities and includes family and friends [37]. Furthermore, PLWHA should be referred for active case management to limit re-infection or non-adherence to medication.
